# Caffeic Acid Cyclohexylamide Rescues Lethal Inflammation in Septic Mice through Inhibition of IκB Kinase in Innate Immune Process

**DOI:** 10.1038/srep41180

**Published:** 2017-02-01

**Authors:** Jun Hyeon Choi, Sun Hong Park, Jae-Kyung Jung, Won-Jea Cho, Byeongwoo Ahn, Cheong-Yong Yun, Yong Pyo Choi, Jong Hun Yeo, Heesoon Lee, Jin Tae Hong, Sang-Bae Han, Youngsoo Kim

**Affiliations:** 1 College of Pharmacy, Chungbuk National University, Cheongju 362-763, Korea; 2 College of Pharmacy, Chonnam National University, Gwangju 500-757, Korea; 3 College of Veterinary Medicine, Chungbuk National University, Cheongju 362-763, Korea

## Abstract

Targeting myeloid differentiation protein 2 (MD-2) or Toll-like receptor 4 (TLR4) with small molecule inhibitor rescues the systemic inflammatory response syndrome (SIRS) in sepsis due to infection with Gram-negative bacteria but not other microbes. Herein, we provided IκB kinase β (IKKβ) in innate immune process as a molecular target of caffeic acid cyclohexylamide (CGA-JK3) in the treatment of polymicrobial TLR agonists-induced lethal inflammation. CGA-JK3 ameliorated *E. coli* lipopolysaccharide (LPS, MD-2/TLR4 agonist)-induced endotoxic shock, cecal ligation and puncture (CLP)-challenged septic shock or LPS plus D-galactosamine (GalN)-induced acute liver failure (ALF) in C57BL/6J mice. As a molecular basis, CGA-JK3 inhibited IKKβ-catalyzed kinase activity in a competitive mechanism with respect to ATP, displaced fluorescent ATP probe from the complex with IKKβ, and docked at the ATP-binding active site on the crystal structure of human IKKβ. Furthermore, CGA-JK3 inhibited IKKβ-catalyzed IκB phosphorylation, which is an axis leading to IκB degradation in the activating pathway of nuclear factor-κB (NF-κB), in macrophages stimulated with TLR (1/2, 2/6, 4, 5, 7, 9) agonists from Gram-positive/negative bacteria and viruses. CGA-JK3 consequently interrupted IKKβ-inducible NF-κB activation and NF-κB-regulated expression of TNF-α, IL-1α or HMGB-1 gene, thereby improving TLRs-associated redundant inflammatory responses in endotoxemia, polymicrobial sepsis and ALF.

Sepsis, a manifestation of SIRS, has been refined as a life-threatening organ dysfunction caused by a dysregulated host response to infection with bacteria most commonly, but also viruses or fungi^1^. Pharmacotherapy of sepsis patients remains elusive. In particular, lipid A derivative Eritoran and non-lipid chemical TAK-242 have completed clinical trials in the treatment of severe sepsis but failed to improve survival rates of sepsis patients; however, the clinical trials recruited patients based on a risk of death but did not consider the etiology of infected pathogens^2^,^3^,^4^. Eritoran antagonizes LPS binding to the receptor MD-2 associated with TLR4, and TAK-242 interacts with the Cys-747 residue on intracellular domain of TLR4, thereby blocking inflammatory responses in sepsis specially due to Gram-negative bacterial infection but not other microbes^5^,^6^. Therefore, novel therapeutic target responding to TLR pathogens from Gram-positive bacteria and viruses, affecting redundant SIRS pathways other than MD-2 or TLR4, is required in the alternative treatment of sepsis patients.

Mammalian TLRs sense not only pathogen-associated molecular patterns from microbes but also danger-associated molecular pattern molecules from dying host cells. They transmit the innate immune responses via intracellular adaptor molecules such as myeloid differentiation factor 88 (MyD88) and toll/IL-1 receptor-containing adaptor inducing interferon (IFN)-β (TRIF)^7^. For immune responses, MyD88 is recruited to several TLRs including TLR1/2, 2/6, 4, 5, 7/8, and 9, while TRIF is specific to TLR3 and 4^7^. TLR/MyD88-dependent pathway stimulates auto-phosphorylation of IL-1 receptor-associated kinase 4 (IRAK-4) and subsequently TGF-β-activating kinase 1 (TAK1)^8^. In turn, TAK1 phosphorylates IKK complex in the activating pathway of NF-κB or stimulates mitogen-activated protein kinases such as JNK and p38 for transcriptional activity of activating protein 1 (AP-1)^9^. Transcription factor NF-κB or AP-1 triggers expression of inflammatory genes encoding TNF-α, IL-1α, HMGB-1 or inducible nitric oxide (NO) synthase (iNOS)^10^. On the other hand, TLR/TRIF-dependent pathway activates transcription factor IRF3 via TNF receptor (TNFR)-associated factor family member-associated NF-κB activator-binding kinase 1 (TBK1), which up-regulates expression of IFN-β or IP-10 gene^11^.

Caffeic acid derivatives are enriched in numerous medicinal plants. They protect from sepsis-related disorders in rodents^12^,^13^,^14^,^15^. In particular, lonicerae flos extract containing caffeic acid quinate (chlorogenic acid) as a major anti-inflammatory constituent is undergoing clinical trial for sepsis treatment. However, their mechanisms remain to be clarified. In the current study, we focused on molecular basis of caffeic acid cyclohexylamide (CGA-JK3, Supplementary Fig. 1A) in the treatment of TLRs-associated redundant inflammatory responses in endotoxemia, polymicrobial sepsis or ALF, and proposed IKKβ inhibition as a potential therapeutic target.

## Results

### CGA-JK3 rescues endotoxic or septic mice

**Figure 1:**
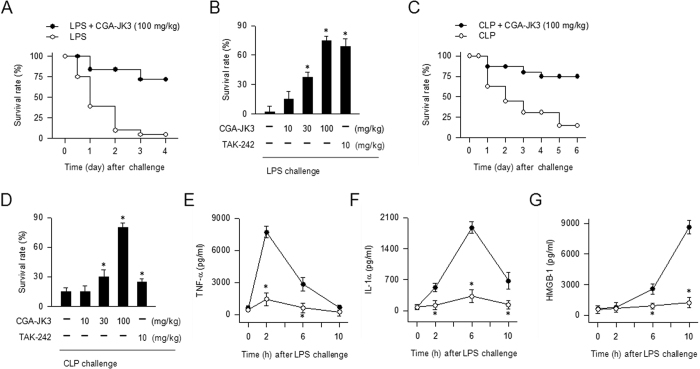
Effects of CGA-JK3 on endotoxemia and sepsis in mice. C57BL/6J mice (each group, n = 9–12) were intraperitoneally injected with LPS (40 mg/kg) for endotoxic shock or challenged with CLP for polymicrobial sepsis, and treated with vehicle, CGA-JK3 or TAK-242 intravenously at 1 h after each intoxication. Survival rates were examined until 4 days after LPS challenge (**A,B**) or 6 days after CLP challenge (**C,D**). Blood samples were collected at 2 h, 6 h or 10 h after LPS challenge, and sera were then loaded onto ELISA kits. Systemic levels TNF-α (**E**), IL-1α (**F**) or HMGB-1 (**G**) are expressed as a solid circle (⦁) in the LPS plus vehicle alone-treated group, and an open circle (о) in the LPS plus CGA-JK3 (100 mg/kg)-treated group. Data are mean ± SEM. *P < 0.05 vs. LPS- or CLP alone-challenged group.

C57BL/6J mice were intraperitoneally (i.p.) injected with LPS (40 mg/kg) for endotoxic shock or challenged with CLP for polymicrobial sepsis, and treated with vehicle, CGA-JK3 or TAK-242 intravenously (i.v.) at 1 h after LPS or CLP challenge. TAK-242, a drug candidate with molecular mechanism of TLR4 inhibition, was used as a positive control agent^3^,^6^. LPS alone-injected mice that developed endotoxic shock were time-dependently sacrificed (Fig. 1A). Treatment with CGA-JK3 decreased mortality rates of endotoxic mice, as did TAK-242 (Fig. 1A,B). CLP alone-challenged mice were time-dependently died due to polymicrobial sepsis (Fig. 1C). The CGA-JK3 (100 mg/kg)-treated group showed 80% survival rate, as compared to <15% survival in the vehicle alone-treated group (Fig. 1C,D), indicating that CGA-JK3 treatment also rescued the septic mice. On the other hand, TAK-242 as a positive control agent showed much lower effectiveness on CLP model than on endotoxemia model (Fig. 1B,D), suggesting different mode of action from CGA-JK3. In endotoxic mice, blood levels of TNF-α increased to maximal values at 2 h after LPS intoxication (Fig. 1E), IL-1α levels at 6 h (Fig. 1F), and HMGB-1 levels at 10 h (Fig. 1G). Treatment with CGA-JK3 attenuated LPS-induced TNF-α, IL-1α or HMGB-1 levels in the blood (Fig. 1E–G), thus improving the cytokine storm *in vivo*.

### CGA-JK3 ameliorates ALF in mice

**Figure 2:**
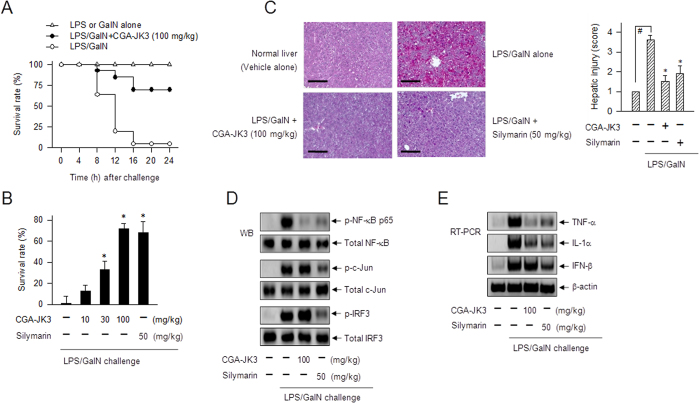
Effect of CGA-JK3 on ALF in mice. C57BL/6J mice (each group, n = 9–12) were intraperitoneally injected with LPS (10 μg/kg) plus GalN (500 mg/kg) for ALF, and treated with vehicle, CGA-JK3 or silymarin intravenously at 1 h after LPS/GalN intoxication. (**A,B**) Survival rates were examined until 24 h after LPS, GalN or LPS/GalN challenge. (**C**) Hepatic lobules were fixed in formalin and embedded in paraffin. The specimens were sectioned serially, and stained with hematoxylin and eosin. The scale bars are 100 μm. Hepatic injury index were scored as described in Methods. Data are mean ± SEM. ^#^*P* < 0.05 vs. vehicle alone-injected group. *P < 0.05 vs. LPS/GalN alone-injected group. (**D**) Cell extracts were prepared from the liver tissues, and subjected to Western blot analysis (WB) with paired antibodies against p-NF-κB p65 and total NF-κB, p-c-Jun and total c-Jun or p-IRF3 and total IRF3. (**E**) Total RNAs were prepared from the liver tissues, and subjected to RT-PCR analysis to determine mRNA levels of TNF-α, IL-1α or IFN-β with β-actin as an internal control.

LPS plus GalN (LPS/GalN)-induced ALF is also known as a TLR-associated lethal disorder with apoptotic death of hepatocytes in the liver, but differs from the multi-organ injury in sepsis or endotoxemia that involves necrosis with minimal apoptosis^16^,^17^. LPS (10 μg/kg)/GalN (500 mg/kg)-injected mice were time-dependently died as a result of ALF, while LPS (10 μg/kg)- or GalN (500 mg/kg) alone-injected mice did not (Fig. 2A). C57BL/6J mice were injected with LPS/GalN (i.p.) for ALF, and treated with vehicle, CGA-JK3 or silymarin (i.v.) at 1 h after LPS/GalN intoxication. Silymarin, a hepatoprotective drug that contains flavolignans, was used as a positive control agent^18^. Treatment with CGA-JK3 or silymarin reduced mortality rates of LPS/GalN-challenged mice with ALF, such that about 70% of the mice survived in the CGA-JK3 (100 mg/kg)- or silymarin (50 mg/kg)-treated group, while most of them died in the vehicle alone-treated group (Fig. 2A,B). LPS/GalN-challenged mice drastically increased aspartate aminotransferase (AST) or bilirubin levels in the blood, which are known as biochemical markers of severe liver injury (Supplementary Fig. 1B,C). Treatment with CGA-JK3 decreased AST or bilirubin levels in the blood of ALF-induced mice (Supplementary Fig. 1B,C). Concurrently, CGA-JK3 or silymarin ameliorated LPS/GalN-induced tissue injury, especially congestion and parenchymal degeneration in the hepatic lobules (Fig. 2C).

To understand whether CGA-JK3 affected TLR-associated immune process, we examined the active indexes via specific phosphorylation of NF-κB p65 at the Ser-536 residue, c-Jun at the Ser-63 residue or IRF3 at the Ser-396 residue^19^,^20^. Treatment with CGA-JK3 decreased phospho (p)-NF-κB p65 levels in the liver with LPS/GalN-induced ALF, but not p-c-Jun and p-IRF3 levels (Fig. 2D). Moreover, treatment with CGA-JK3 suppressed mRNA levels of NF-κB-target genes encoding TNF-α and IL-1α in the liver, but not those of IRF3-target IFN-β gene (Fig. 2E). On the other hand, silymarin as a positive control agent inhibited LPS/GalN-induced phosphorylation of c-Jun or IRF3 in addition to NF-κB p65, and suppressed mRNA levels of TNF-α, IL-1α and IFN-β (Fig. 2D,E), suggesting different mechanism from CGA-JK3.

### CGA-JK3 inhibits IκBα phosphorylation in macrophages

**Figure 3:**
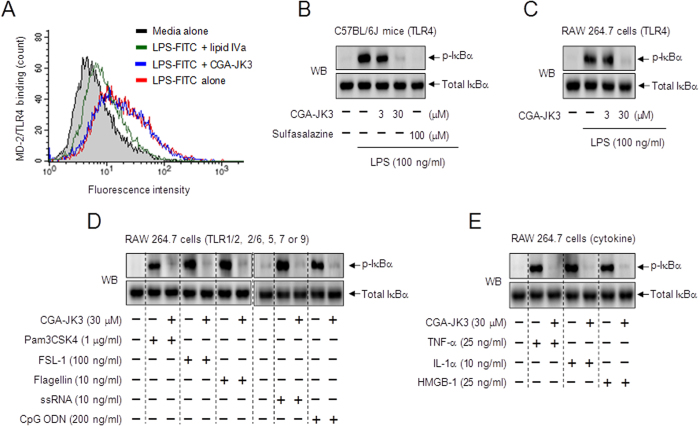
Effect of CGA-JK3 on IκBα phosphorylation. (**A**) RAW 264.7 cells were incubated with LPS-FITC (1 μg/ml) for 30 min in the presence of CGA-JK3 (30 μM) or lipid IVa (100 nM). After washing, the cells were subjected to flow cytometric analysis. Mouse peritoneal macrophages (**B**) or RAW 264.7 cells (**C**) were pretreated with CGA-JK3 for 2 h and stimulated with LPS for 10–20 min in the presence of CGA-JK3. RAW 264.7 cells were pretreated with CGA-JK3 for 2 h and stimulated with TLR agonists (**D**) or cytokines (**E**) for 10–20 min in the presence of CGA-JK3. Cell extracts were subjected to Western blot analysis (WB) with anti-p-IκBα or anti-IκBα antibody.

CGA-JK3 showed no effect on binding of fluorescent LPS probe (LPS-FITC) to extracellular MD-2 associated with transmembrane TLR4 in macrophages, whereas lipid IVa (MD-2 antagonist) resulted in inhibition of the binding (Fig. 3A), thus excluding the possibility of direct effect of CGA-JK3 on the receptor or LPS scavenging. To elucidate a molecular mechanism of CGA-JK3, we examined the phosphorylation of IκBα at the Ser-32 and Ser-36 residues, a cellular substrate of IKKβ, because it decreased protein or mRNA levels of NF-κB-target genes *in vivo* (Figs 1E–G and 2D,E). CGA-JK3 inhibited LPS-induced IκBα phosphorylation in mouse peritoneal macrophages or RAW 264.7 monocytic cells (Fig. 3B,C). CGA-JK3 also inhibited Pam3CSK4 (TLR1/2 agonist mimicking the triacylated lipoprotein from Gram-positive bacteria)-, FSL-1 (TLR2/6 agonist mimicking the diacylated lipoprotein from *Mycoplasma fermentans*)-, flagellin (TLR5 agonist from bacterial flagellar filament)-, ssRNA (TLR7 agonist mimicking the viral RNAs)- or CpG ODN (TLR9 agonist mimicking the bacterial or viral unmethylated CpG DNA)-induced IκBα phosphorylation in RAW 264.7 cells (Fig. 3D). Furthermore, CGA-JK3 inhibited TNF-α-, IL-1α- or HMGB-1-induced IκBα phosphorylation in the cells (Fig. 3E).

However, CGA-JK3 showed no effect on the Pam3CSK4- or LPS-stimulated auto-phosphorylation of IRAK-4 at the Thr-345 and Ser-346 residues in RAW 264.7 cells or the TNF-α- or LPS-stimulated auto-phosphorylation of TAK1 at the Thr-184 and Thr-187 residues, in which IS409 (IRAK-1/4 inhibitor) and LLZ 1640–2 (TAK1 inhibitor) were used as positive control agents (Supplementary Fig. 2A,B). CGA-JK3 at concentrations up to 30 μM did not disturb the viability of RAW 264.7 cells (Supplementary Fig. 2C), excluding the possibility of nonspecific cytotoxicity. The results suggested that CGA-JK3 inhibited IKKβ-catalyzed IκBα phosphorylation in macrophages stimulated with various TLR agonists from bacteria and viruses or with endogenous cytokines, while it did not affect TLR- or TNFR-recruited early signal transducers, including IRAK-4 and TAK1, that are located upstream from IKKβ.

### CGA-JK3 is an ATP-competitive inhibitor of IKKβ-catalyzed kinase activity

**Figure 4:**
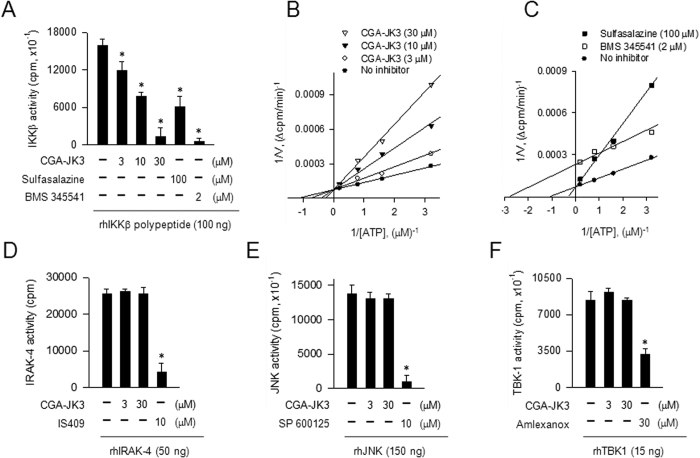
Effect of CGA-JK3 on IKKβ-catalyzed kinase activity. Catalytically active rhIKKβ (**A–C**), rhIRAK-4 (**D**), rhJNK (**E**) or rhTBK1 (**F**) was treated with CGA-JK3 for 10 min in cell-free reactions. *In vitro* kinase assay was then monitored by incorporation of [^32^P] from the probe [γ-^32^P]ATP onto IKKtide (**A–C**) or MBP (**D–F**) as exogenous substrate. Data are mean ± SEM from three independent experiments using the average values of triplicate in each experiment. *P < 0.05 vs. rhIKKβ- or other protein kinase alone-containing group. (**B,C**) Kinetic data of rhIKKβ-catalyzed kinase activity are represented as mean values of 1/V, an inverse of the initial increase of cpm values per min (Δcpm/min), from three independent experiments with varying concentrations of ATP.

To understand whether CGA-JK3 directly inhibited the kinase activity of IKKβ, catalytically active rhIKKβ was treated with CGA-JK3 in cell-free reactions and then reacted with IKK substrate peptide (IKKtide) in the presence of [γ-^32^P]ATP probe. CGA-JK3 dose-dependently inhibited the rhIKKβ-catalyzed kinase activity, as did sulfasalazine and BMS 345541 as positive control agents (Fig. 4A). Sulfasalazine, a FDA-approved drug for rheumatoid arthritis or inflammatory bowel disease with chronic inflammation, is an ATP-competitive inhibitor of IKKβ activity, and BMS 345541 is an allosteric inhibitor^21^,^22^. In a kinetic study, rhIKKβ exhibited a *K*_*m*_ value of 0.93 μM and a *V*_*max*_ value of 13,400 Δcpm/min with varying concentrations of ATP (Fig. 4B,C). Treatment with CGA-JK3 increased the *K*_*m*_ value but did not alter the *V*_*max*_ value of IKKβ-catalyzed kinase activity (Fig. 4B), suggesting a competitive mechanism with respect to ATP. Sulfasalazine as a positive control agent also increased the *K*_*m*_ value without altering the *V*_*max*_ value, as did CGA-JK3, but BMS 345541 changed both *K*_*m*_ and *V*_*max*_ values (Fig. 4C). However, CGA-JK3 did not inhibit the kinase activities of cell-free rhIRAK-4, rhJNK or rhTBK1 that are also associated with TLR-dependent immune process, in which IS409, SP 600125 and amlexanox were used as positive control agents (Fig. 4D–F). SP 600125 is an ATP-competitive inhibitor of JNK activity and amlexanox is an ATP-competitive inhibitor of TBK1 activity^23^,^24^.

**Figure 5:**
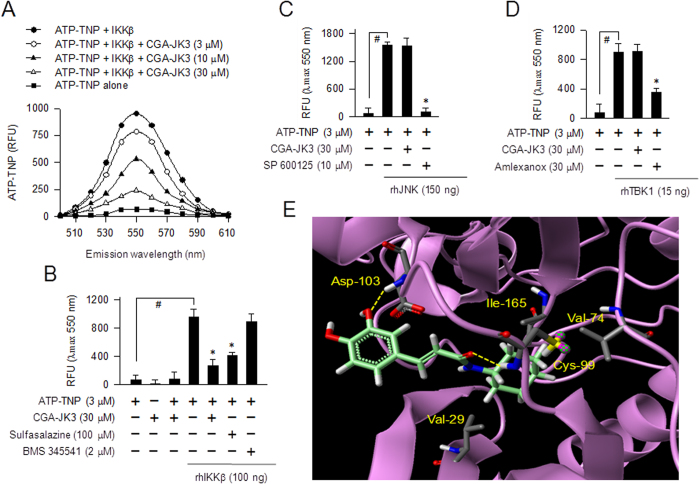
Effect of CGA-JK3 on ATP-TNP binding to IKKβ. (**A**) ATP-TNP (3 μM) was pre-incubated with rhIKKβ (100 ng) for 2 h in cell-free reactions to achieve stable fluorescence values under excitation at 400 nm, and then treated with CGA-JK3 for another 2 h. Emission spectra at 500–610 nm are represented as relative fluorescence units (RFU). ATP-TNP was pre-incubated with rhIKKβ (**B**), rhJNK (**C**) or rhTBK1 (**D**) for 2 h in cell-free reactions and then treated with CGA-JK3 for another 2 h. Fluorescence values are represented as RFU under excitation at 400 nm and emission at 550 nm. Data are mean ± SEM from three independent experiments using the average values of triplicate in each experiment. ^#^*P* < 0.05 vs. ATP-TNP alone-containing group. *P < 0.05 vs. ATP-TNP plus rhIKKβ- or ATP-TNP plus other protein kinase alone-containing group. (**E**) Docking arrangement of CGA-JK3 to the crystal structure of human IKKβ was carried out with the Surflex-Dock program. CGA-JK3 is represented as a green color, catalytic residues on the ATP-binding active site of IKKβ as a grey color, and the other IKKβ backbone as a violet color. Hydrogen bonding between CGA-JK3 and IKKβ is indicated as a yellow dotted line.

To clarify whether CGA-JK3 interacted with the ATP-binding site of IKKβ, fluorescent ATP probe (2′,3′-*O*-(2,4,6-trinitrophenyl)adenosine triphosphate, ATP-TNP) was pre-incubated with rhIKKβ in cell-free reactions to achieve stable fluorescence intensity, and then treated with CGA-JK3. The fluorescence intensity of ATP-TNP was markedly increased following its binding to rhIKKβ (Fig. 5A). Post-treatment with CGA-JK3 dose-dependently decreased rhIKKβ-enhanced fluorescence values of ATP-TNP (Fig. 5A), indicating displacement of ATP-TNP from the complex with rhIKKβ. However, non-fluorescent CGA-JK3 did not alter basal fluorescence values of ATP-TNP in the absence of rhIKKβ (Fig. 5B). Consistent with *in vitro* kinase assays (Fig. 4E,F), CGA-JK3 did not affect ATP-TNP binding to rhJNK or rhTBK1, in which SP 600125 and amlexanox were used as positive control agents (Fig. 5C,D).

Based on the evidences showing that CGA-JK3 inhibited the kinase activity of IKKβ in a competitive mechanism with respect to ATP and displaced fluorescent ATP probe from the complex with IKKβ, we conducted molecular docking with the crystal structure of human IKKβ^25^. CGA-JK3 was well fitted into the ATP-binding active site of IKKβ with close contacts to the Val-29, Val-74, Cys-99, Asp-103 and Ile-165 residues under the most energetically favorable simulation (Fig. 5E). Hydrogen bonding was achieved between the amide carbonyl group of CGA-JK3 and the peptidyl amino backbone of Cys-99 residue, and between the phenolic hydroxyl group of CGA-JK3 and the peptidyl amino backbone of Asp-103 residue (Fig. 5E). The cyclohexyl moiety of CGA-JK3 was exposed to a hydrophobic environment consisting with Val-29, Val-74 and Ile-165 (Fig. 5E). Moreover, the docking simulation of CGA-JK3 overlapped with that of endogenous ligand, ATP bound to IKKβ (Supplementary Fig. 3).

### CGA-JK3 suppresses the transcriptional activity of NF-κB but not AP-1 or IRF3

**Figure 6:**
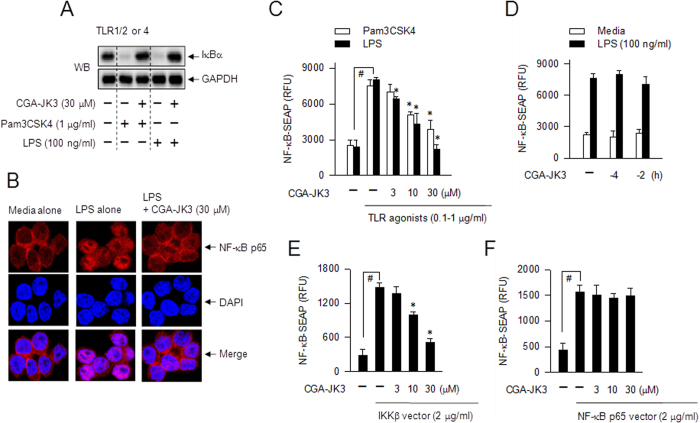
Effect of CGA-JK3 on NF-κB activating pathway. RAW 264.7 cells were pretreated with CGA-JK3 for 2 h, and stimulated with Pam3CSK4 or LPS for 30–40 min (**A**) or with LPS (100 ng/ml) for 1 h (**B**) in the presence of CGA-JK3. (**A**) Cell extracts were subjected to Western blot analysis (WB) with anti-IκBα or anti-GAPDH antibody. (**B**) The cells were subjected to confocal fluorescence microscopy, displaying the NF-κB p65-stained with Alexa Fluor 568-labeled antibody as a red color and the nuclei-stained with DAPI as a blue color. (**C**) RAW 264.7 cells harboring NF-κB-SEAP reporter construct were stimulated with Pam3CSK4 (1 μg/ml) or LPS (100 ng/ml) for 20 h in the presence of CGA-JK3. (**D**) RAW 264.7 cells harboring NF-κB-SEAP reporter construct were treated with CGA-JK3 for 2–4 h, washed with PBS and recovered in complete media. The cells were then stimulated with LPS for 20 h. RAW 264.7 cells harboring NF-κB-SEAP reporter construct were transfected with expression vector encoding IKKβ (**E**) or NF-κB p65 (**F**) in combination with β-galactosidase control vector. The transfected cells were treated with CGA-JK3 for 20 h. SEAP activity, a reporter of NF-κB transcriptional activity, is represented as relative fluorescence units (RFU) (**C,D**) or RFU after normalizing to the β-galactosidase activity as a reference of transfection efficiency (**E,F**). Data are mean ± SEM from three independent experiments using the average values of triplicate in each experiment. ^#^*P* < 0.05 vs. media alone-added group. *P < 0.05 vs. Pam3CSK4- or LPS alone-stimulated group (**C**) or IKKβ vector alone-transfected group (**E**).

In Pam3CSK4- or LPS-activated RAW 264.7 cells, treatment with CGA-JK3 sequentially inhibited IκBα degradation (Fig. 6A), nuclear import of NF-κB p65 (Fig. 6B), and transcriptional activity of NF-κB (Fig. 6C), which are located downstream from IKKβ-catalyzed IκBα phosphorylation in the NF-κB activating pathway^10^. To determine whether the effect of CGA-JK3 on NF-κB activation was reversible. RAW 264.7 cells harboring NF-κB-secretory alkaline phosphatase (SEAP) construct, a reporter of NF-κB transcriptional activity, were pre-incubated with CGA-JK3 for 2–4 h, allowed to recover in complete media after washing, and then stimulated with LPS. CGA-JK3 did not affect LPS-induced NF-κB transcriptional activity when it was washed out before LPS stimulation (Fig. 6D), suggesting a reversible mechanism of action. To determine whether IKKβ was a primary target of CGA-JK3 in the suppression of NF-κB activating pathway, we transfected RAW 264.7 cells harboring NF-κB-SEAP reporter construct with expression vector encoding IKKβ or NF-κB p65. The ectopic expression of IKKβ or NF-κB p65, which bypasses TLRs or cytokine receptors, increased SEAP activity as a reporter of NF-κB transcriptional activity (Fig. 6E,F). CGA-JK3 decreased IKKβ vector-elicited NF-κB transcriptional activity (Fig. 6E), but not SEAP activity under the control of NF-κB p65 vector (Fig. 6F). This result excluded the possibility of direct effect of CGA-JK3 on the NF-κB activating pathway that is located downstream from IKKβ.

However, CGA-JK3 showed no inhibition of the c-Jun phosphorylation at the Ser-63 residue in LPS- or CpG ODN-activated RAW 264.7 cells, and the AP-1 transcriptional activity in LPS-activated cells containing AP-1-Luc reporter construct (Supplementary Fig. 4A,B). In addition, CGA-JK3 did not affect the IRF3 phosphorylation at the Ser-396 residue in poly I:C- or LPS-activated RAW 264.7 cells, and the IRF3 transcriptional activity in LPS-activated cells containing IRF3-Luc reporter construct (Supplementary Fig. 4C,D). The results suggested that CGA-JK3 interrupted IKKβ-inducible NF-κκB activation in macrophages, whereas it had no effects on TLR/MyD88-associated AP-1 activation and TLR/TRIF-dependent IRF3 activation.

**Figure 7:**
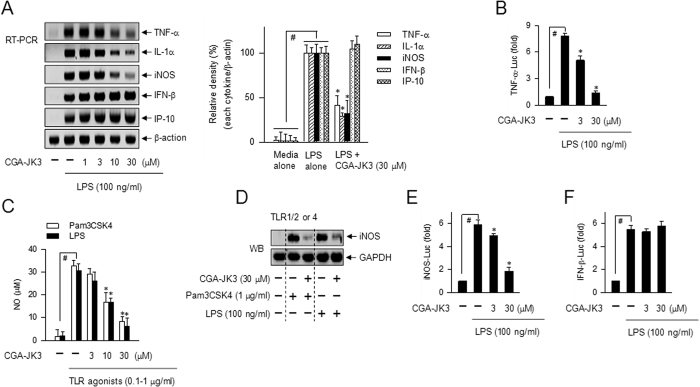
Effect of CGA-JK3 on NF-κB-regulated gene expression. (**A**) RAW 264.7 cells were pretreated with CGA-JK3 for 2 h and stimulated with LPS for 4–6 h in the presence of CGA-JK3. Total RNAs were subjected to RT-PCR analysis to determine mRNA levels of TNF-α, IL-1α, iNOS, IFN-β or IP-10 with β-actin as an internal control. Relative intensity of each cytokine normalized to β-actin is also represented as %. RAW 264.7 cells were transfected with each reporter construct of TNF-α (−1260/+60)-Luc (**B**), iNOS (−1592/+183)-Luc (**E**) or IFN-β (−1814/+11)-Luc (**F**) in the combination with *Renilla* control vector. The transfected cells were stimulated with LPS for 20 h in the presence of CGA-JK3. Cell extracts were subjected to dual-luciferase assay. Firefly luciferase activity, a reporter of the promoter activity of TNF-α, iNOS or IFN-β gene, is represented as relative fold after normalizing to the *Renilla* activity as a reference of transfection efficiency. RAW 264.7 cells were stimulated with Pam3CSK4 (1 μg/ml) or LPS (100 ng/ml) for 24 h in the presence of CGA-JK3. (**C**) Aliquots of the culture supernatants were reacted with Griess reagents to determine NO levels with NaNO_2_ as a standard. (**D**) Cell extracts were subjected to Western blot analysis (WB) with anti-iNOS or anti-GAPDH antibody. Data are mean ± SEM from three independent experiments using the average values of triplicate in each experiment. ^#^*P* < 0.05 vs. media alone-added group. *P < 0.05 vs. LPS- or Pam3CSK4 alone-stimulated group.

CGA-JK3 suppressed mRNA and protein levels of TNF-α or IL-1α in LPS-activated macrophages (Fig. 7A and Table 1), which was consistent with the *in vivo* effects in endotoxemia- or ALF-induced mice (Figs 1E,F and 2E). RAW 264.7 cells were then transfected with TNF-α-Luc reporter construct containing TNF-α promoter region (−1260/+60). CGA-JK3 inhibited LPS-induced promoter activity of TNF-α gene (Fig. 7B). Moreover, CGA-JK3 inhibited NO production and decreased protein levels of iNOS in Pam3CSK4- or LPS-activated RAW 264.7 cells (Fig. 7C,D). CGA-JK3 consistently attenuated mRNA levels of iNOS in LPS-activated RAW 264.7 cells (Fig. 7A), and inhibited promoter activity of iNOS gene in LPS-activated cells containing iNOS (−1592/+183)-Luc reporter construct (Fig. 7E). The results suggested that CGA-JK3 suppressed NF-κB-regulated expression of TNF-α, IL-1α or iNOS gene at the transcription level. However, CGA-JK3 affected neither the mRNA and protein levels of IRF3-target genes encoding IFN-β or IP-10 in LPS-activated macrophages (Fig. 7A and Table 1), nor the promoter activity of IFN-β gene in LPS-activated cells containing IFN-β (−1814/+11)-Luc reporter construct (Fig. 7F).

## Discussion

In the current study, CGA-JK3 ameliorated TLRs-associated endotoxemia, polymicrobial sepsis or ALF *in vivo*. As a molecular mechanism, CGA-JK3 targeted the ATP-binding active site of IKKβ on the basis of evidences showing that it inhibited IKKβ-catalyzed kinase activity in a competitive manner with respect to ATP, and displaced fluorescent ATP probe from the complex with IKKβ.

The pathophysiological importance of IKKβ has been revealed in gene knockout (KO) mice. Classical KO mice lacking IKKβ die at an embryonic stage due to severe liver degeneration, but can be rescued after TNFR inactivation^26^,^27^. On the other hand, conditional KO mice with parenchymal hepatocyte-specific deletion of IKKβ, which bypass the embryonic lethality, are attenuated from soluble TNF-α-induced apoptotic injury and ischemia/reperfusion-induced necrotic damage in the liver^28^,^29^. Therefore, it is likely that IKKβ has differential roles in the liver during the embryonic development and adult stages.

Caffeic acid derivatives from medicinal plants have numerous anti-inflammatory benefits. In particular, caffeic acid quinate (chlorogenic acid) rescues endotoxemia-induced mice via directly inhibiting IRAK-4-catalyzed kinase activity, and CLP-induced septic mice via decreasing TNF-α or HMGB-1 levels^13^,^14^,^15^. Caffeic acid phenethyl ester (CAPE) ameliorates LPS-induced endotoxemia in rats by correcting the imbalance between pro- and anti-inflammatory cytokines in the blood, and reduces mortality rates of CLP-challenged septic mice^12^,^30^. CAPE also protects from LPS/GalN-induced ALF in rats with restored antioxidant defense in the liver^31^. CAPE has a negative regulatory role in the NF-κB activating pathway, but its molecular target is controversially assigned as either DNA-binding ability of NF-κB or unidentified signal transducer(s) upstream from IκB phosphorylation/degradation^32^,^33^,^34^. In the current study, chlorogenic acid inhibited the kinase activity of rhIRAK-4 but not rhIKKβ in cell-free reactions, whereas CAPE did not affect the kinase activities of both rhIRAK-4 and rhIKKβ (Supplementary Fig. 5A,B), suggesting different molecular targets from CGA-JK3.

In conclusion, CGA-JK3 interrupted IKKβ-inducible NF-κB activation and NF-κB-regulated gene expression in macrophages stimulated with TLR (1/2, 2/6, 4, 5, 7, 9) agonists from Gram-positive/negative bacteria and viruses or with endogenous cytokines (TNF-α, IL-1α, HMGB-1). Thus, IKKβ was a potential molecular target of CGA-JK3 in the amelioration of TLRs-associated redundant inflammatory responses in endotoxemia-, sepsis- or ALF-induced mice. Finally, CGA-JK3 may be more attractable in the treatment of polymicrobes-infected septic disorders than the drug candidates Eritoran and TAK-242 that are limiting to inflammatory responses in Gram-negative bacteria-infected sepsis. CGA-JK3, a small molecule inhibitor of IKKβ in TLRs-associated immune responses, may be beneficial and reduce mortality of sepsis patients in whom inflammation is excessive and itself causes injury but may harm to the patients in immune suppressive stages, since sepsis leads to high morbidity and mortality through a complex pathophysiology including SIRS, compensatory anti-inflammatory response syndrome, and abnormal blood coagulation^35^,^36^,^37^,^38^. Further study relating to safety and efficacy profiles of CGA-JK3 or other IKKβ inhibitors would be necessary to translate these concepts into clinical application.

## Methods

### Chemicals and antibodies

CGA-JK3 (>97% purity) was synthesized from caffeic acid by reacting with cyclohexylamine under an amidation condition. rhIKKβ or other protein kinases were purchased from SignalChem (Richimond, Canada), TLR agonists from Invitrogen (Carlsbad, CA, USA), ATP-TNP from Life Technology (Bangalore, India), and IKKtide or myeloid basic protein (MBP) from Millipore (Temecula, CA, USA). Antibodies were purchased from Santa Cruz Biotechnology (Santa Cruz, CA, USA) and Cell Signaling Technology (Danvers, MA, USA). All other materials, including LPS-FITC, were purchased from Sigma-Aldrich (St. Louis, MO, USA).

### Sepsis or ALF model in mice

C57BL/6J male mice, aged 6–7 weeks, were purchased from Korea Research Institute of Bioscience and Biotechnology (Cheongju, Korea), and kept under well-controlled conditions of temperature (22 ± 2 °C), humidity (55 ± 5%) and a 12 h/12 h light-dark cycle with access to food and water ad libitum. The protocol was approved by the Animal Experimentation Ethics Committee in Chungbuk National University (permit number CBNUA-809–15–01), and conducted in accordance with the Korean Ministry of Food and Drug Safety Guide for the Care and Use of Laboratory Animals. C57BL/6J mice were challenged with LPS (40 mg/kg, i.p.) for endotoxemia, CLP for polymicrobial sepsis, or LPS (10 μg/kg, i.p.)/GalN (500 mg/kg, i.p.) for ALF, and treated with CGA-JK3 (i.v.) at 1 h after each challenge. Survival rates were examined as primary outcome. Blood samples were collected to analyze systemic levels of AST, bilirubin or cytokines. Hepatic lobules were fixed in 10% formalin and embedded in paraffin. Serial sections (3 μm thick) of the specimens were stained with hematoxylin and eosin for histological examination, and hepatic injury index was scored as normal = 1, mild = 2, moderate = 3, and severe = 4.

### Western blot analysis

Cell extracts were resolved on SDS-acrylamide gels by electrophoresis and transferred to polyvinylidene difluoride membranes. Blocking was with either 5% non-fat milk in PBS containing Tween 20 or 5% BSA in TBS containing Tween 20. The blots were incubated with primary antibody at 4 °C overnight followed by the appropriate horseradish peroxidase-labeled secondary antibody at room temperature for 3–5 h. The immune complex was visualized after reacting with an enhanced chemiluminescence kit (GE Healthcare, Chalfont St. Giles, UK).

### RT-PCR analysis

Total RNAs were subjected to RT-PCR with an RNA PCR kit (Bioneer, Daejeon, Korea) in the determination of mRNA levels of TNF-α, IL-1α, IFN-β, iNOS or IP-10. Nucleotide sequences of the PCR primers were previously described^15^. In brief, total RNAs were reversely transcribed at 42 °C for 1 h and then subjected to 25–30 cycles of PCR. Cycling conditions were 30-s denaturation at 94 °C, 60-s annealing at 50–60 °C and 90-s extension at 72 °C. The RT-PCR products were resolved on agarose gels by electrophoresis and stained with ethidium bromide.

### Cell culture

RAW 264.7 cells were purchased from ATCC (Manassas, VA, USA), and peritoneal macrophages were isolated from C57BL/6J mice. In brief, mice were anesthetized with diethyl ether inhalation and the abdominal area was disinfected with 70% ethyl alcohol. Peritoneal cavities of the mice were flushed with ice-cold PBS to harvest macrophages. RAW 264.7 cells or peritoneal macrophages were cultured in Dulbecco’s modified Eagle’s medium supplemented with 10% fetal bovine serum, benzylpenicillin (143 U/ml) and streptomycin (100 μg/ml) under an atmosphere of 37 °C and 5% CO_2_.

### Flow cytometry

Cells were incubated with LPS-FITC (500 ng/ml) for 30 min. Flow cytometric analysis was then conducted using FACSCalibur (BD Bioscience, San Jose, CA, USA).

### Cell viability assay

Cells were reacted with 3-(4,5-dimethylthiazol-2-yl)-2,5-diphenyltetrazolium bromide (MTT, 50 μg/ml) for 2–4 h. Formazan crystals were dissolved in 50% dimethyl sulfoxide and absorbance was measured at 540 nm.

### Protein kinase assay

Catalytically active rhIKKβ or other protein kinases were reacted with IKKtide (1 mg/ml) or MBP (0.33 mg/ml) as exogenous substrate in the presence of [γ-^32^P]ATP (5 μCi) probe at 30 °C for 30 min. Aliquots of the reaction mixtures were spotted onto P81 phosphocellulose, and washed three times with 0.75% H_3_PO_4_ followed by once with 100% acetone. Radioactivity was measured as count per min (cpm). Lineweaver-Burk plots were used to estimate kinetic parameters, including *K*_*m*_ and *V*_*max*_, of rhIKKβ-catalyzed kinase activity.

### Fluorescence analysis

ATP-TNP (3 μM) was incubated with rhIKKβ or other protein kinases for 2 h. Fluorescence values were measured as relative fluorescence units (RFU) under excitation at 400 nm and emission at 500–610 nm.

### Molecular docking

Crystallographic structure of human IKKβ was obtained from the Protein Data Bank (PDB code 3RZF). Chemical structure of CGA-JK3 was drawn with the Sybyl package and minimized with Tripos force field and Gasteiger-Huckel charge. Docking arrangement of CGA-JK3 onto the crystal structure of IKKβ was carried out with the Surflex-Dock program in Sybyl version 8.1.1 (Tripos Associates, St. Louis, MO, USA).

### Confocal microscopy

Cells were fixed in 4% *p*-formaldehyde, permeabilized in 0.5% Triton X-100 and then blocked in 1% BSA. The cells were reacted with anti-NF-κB p65 antibody followed by Alexa Fluor 568-labeled secondary antibody for immunostaining, incubated with 4,6-diamidino-2-phenylindole (DAPI, 3 μM) for nuclei staining, and then examined under confocal fluorescence microscopy.

### SEAP reporter assay

RAW 264.7 cells harboring NF-κB-SEAP reporter construct were used in the determination of NF-κB transcriptional activity^15^,^39^. Aliquots of the culture supernatants were heated at 65 °C for 5 min and then reacted with 4-methylumbelliferyl phosphate (500 μM) in the dark. SEAP activity was measured as RFU under excitation at 360 nm and emission at 450 nm.

### Luciferase reporter assay

Luciferase reporter constructs of AP-1-Luc or GAS/ISRE-Luc were used in the determination of transcriptional activity of AP-1 or IRF3 (Promega, Madison, WI, USA), and those of TNF-α (−1260/+60)-Luc, iNOS (−1592/+183)-Luc or IFN-β (−1814/+11)-Luc for the promoter activity of TNF-α, iNOS or IFN-β gene^40^,^41^. Cells were transfected with each reporter construct in combination with *Renilla* control vector using a lipofectamine kit (Invitrogen, Carlsbad, CA, USA). Cell extracts were subjected to dual-luciferase assay (Promega, Madison, WI, USA). Firefly luciferase activity as the reporter was normalized to *Renilla* activity as a reference for transfection efficiency.

### NO quantification

Supernatants from macrophage cultures were reacted with Griess reagents (0.1% sulfanilamide and 0.1% *N*-(1-naphthyl)ethylenediamine in 5% H_3_PO_4_), and absorbance was measured at 540 nm with NaNO_2_ as a standard.

### Statistical analysis

Results were expressed as mean ± SEM from at least three independent experiments. Data were statistically analyzed using Dunnett’s test in the ANOVA. Values of *P* < 0.05 were considered significant.

## Additional Information

**How to cite this article**: Choi, J. H. *et al*. Caffeic Acid Cyclohexylamide Rescues Lethal Inflammation in Septic Mice through Inhibition of IκB Kinase in Innate Immune Process. *Sci. Rep.* **7**, 41180; doi: 10.1038/srep41180 (2017).

**Publisher's note:** Springer Nature remains neutral with regard to jurisdictional claims in published maps and institutional affiliations.

## Supplementary Material

Supplementary Information

## Figures and Tables

**Table 1:** Effect of CGA-JK3 on LPS-induced production of TNF-α, IL-1α or IFN-β in macrophages.

Treatment	TNF-α (pg/ml)	IL-1α (pg/ml)	IFN-β (pg/ml)
None	306 ± 87	43 ± 15	117 ± 29
LPS alone	1867 ± 104^#^	429 ± 46^#^	735 ± 68^#^
LPS + CGA-JK3 (1 μM)	1704 ± 161	375 ± 27	732 ± 54
LPS + CGA-JK3 (3 μM)	1430 ± 96*	353 ± 51	701 ± 22
LPS + CGA-JK3 (10 μM)	1005 ± 125*	226 ± 38*	818 ± 54
LPS + CGA-JK3 (30 μM)	415 ± 79*	125 ± 26*	738 ± 47

Peritoneal macrophages were isolated from C57BL/6 J mice. The cells were stimulated with LPS (100 ng/ml) for 24 h in the presence of CGA-JK3. Aliquots of the culture supernatants were loaded onto ELISA kits to determine protein levels of TNF-α, IL-1α or IFN-β. Data are mean ± SEM from three independent experiments using the average values of triplicate in each experiment. ^#^*P* < 0.05 vs. media alone-added group. *P < 0.05 vs. LPS alone-stimulated group.
