# Supplementary material for: Caffeic Acid Cyclohexylamide Rescues Lethal Inflammation in Septic Mice through Inhibition of IκB Kinase in Innate Immune Process
Source: Sci Rep. 2017 Feb 1;7:41180. doi: 10.1038/srep41180 (PMC5286524; doi:10.1038/srep41180)
Supplement: Supplementary Information [file srep41180-s1.pdf]

Supplementary Information

Title: Caffeic Acid Cyclohexylamide Rescues Lethal Inflammation in Septic Mice through Inhibition of IκB Kinase in Innate Immune Process

Authors: Jun Hyeon Choi[1], Sun Hong Park[1], Jae-Kyung Jung[1], Won-Jea Cho[2], Byeongwoo Ahn[3], Cheong-Yong Yun[1], Yong Pyo Choi[1], Jong Hun Yeo[1], Heesoon Lee[1], Jin Tae Hong[1], Sang-Bae Han[1] & Youngsoo Kim[1,*]

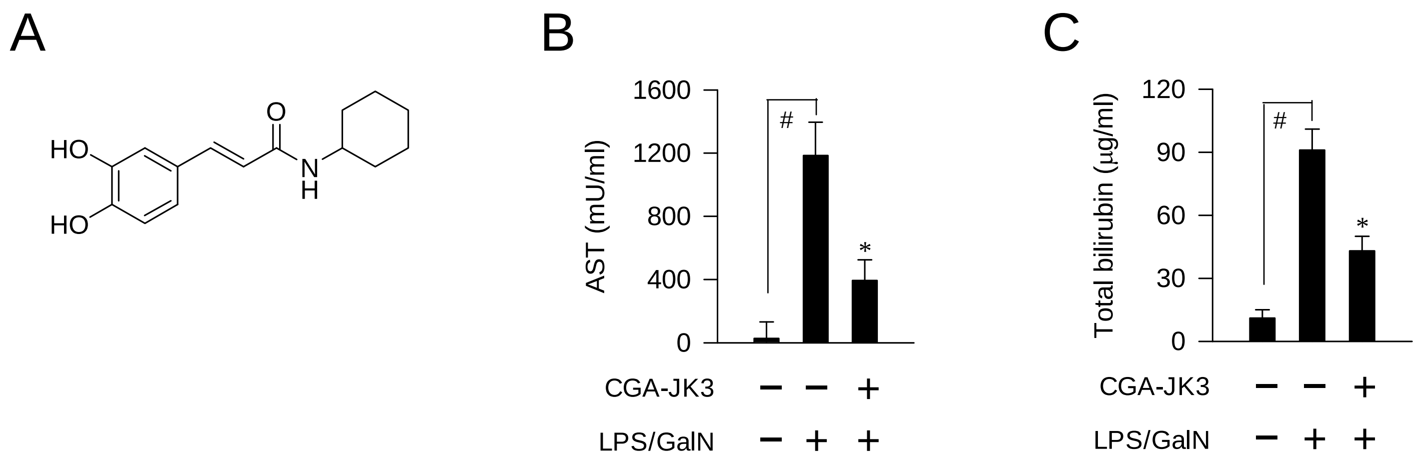

Supplementary Figure 1. Effect of CGA-JK3 on AST or bilirubin levels in the blood. (**A**) Chemical structure of CGA-JK3. (**B, C**) C57BL/6J mice (each group, n = 5) were intraperitoneally injected with LPS (10 μg/kg)/GalN (500 mg/kg) for ALF and treated with vehicle or CGA-JK3 (100 mg/kg) intravenously at 1 h after LPS/GalN challenge. Blood samples were collected to determine AST or bilirubin levels. Data are mean ± SEM. $^{\#}P < 0.05$ vs. vehicle alone-injected group. $^{*}P < 0.05$ vs. LPS/GalN alone-injected group.

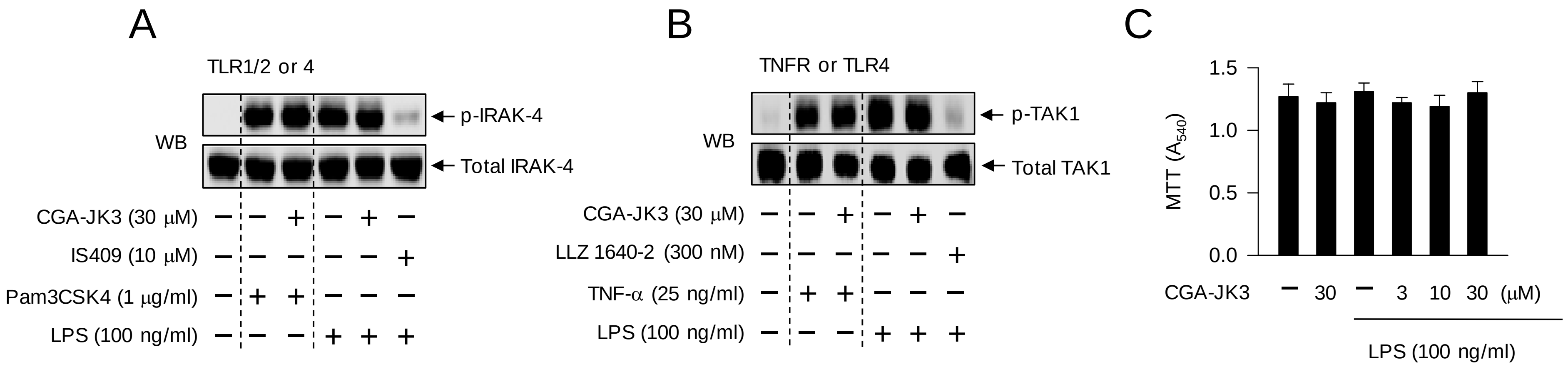

Supplementary Figure 2. Effect of CGA-JK3 on the auto-phosphorylation of IRAK-4 or TAK1. RAW 264.7 cells were pretreated with CGA-JK3 for 2 h, and stimulated with Pam3CSK4 or LPS for 5-10 min (**A**) and TNF-α or LPS for 10-20 min (**B**) in the presence of CGA-JK3. Cell extracts were subjected to Western blot analysis (WB) with paired antibodies against p-IRAK-4 and total IRAK-4 (**A**) or p-TAK1 and total TAK1 (**B**). (**C**) RAW 264.7 cells were incubated with CGA-JK3 for 24 h in the absence or presence of LPS. Cell viability was measured by MTT assay, and is represented as absorbance values at 540 nm (A_{540}). Data are mean ± SEM from three independent experiments using the average values of triplicate in each experiment.

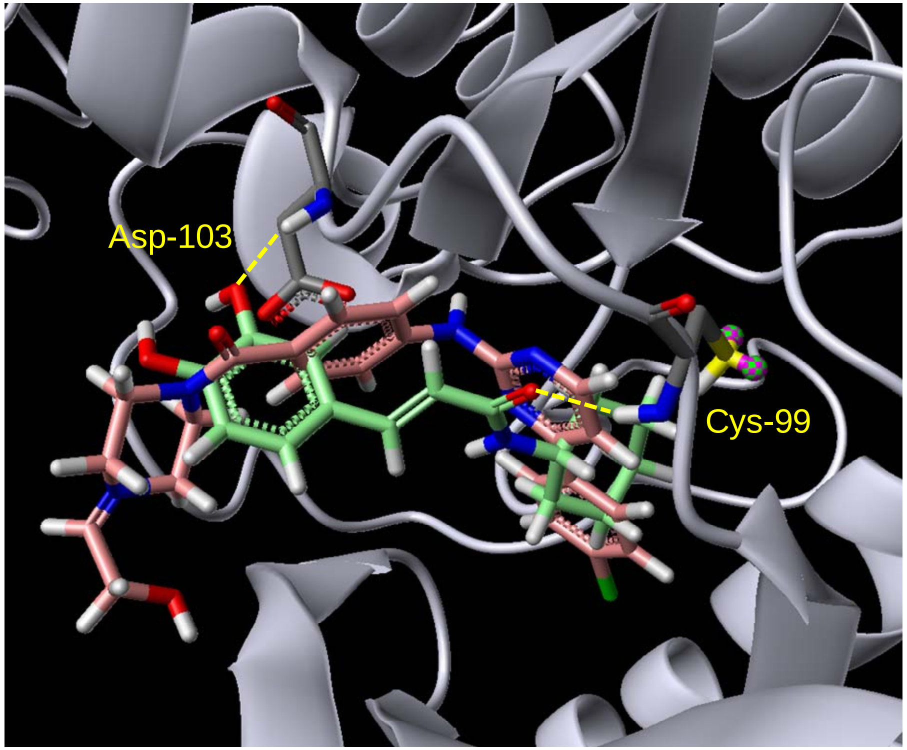

Supplementary Figure 3. Superimposed docking of CGA-JK3 onto ATP bound to IKKβ. Docking arrangement of CGA-JK3 to the crystal structure of human IKKβ was carried out with the Surflex-Dock program, and then superimposed onto that of endogenous ligand, ATP bound to IKKβ. CGA-JK3 is represented as a green color, ATP as a pink color, and IKKβ as a grey color.

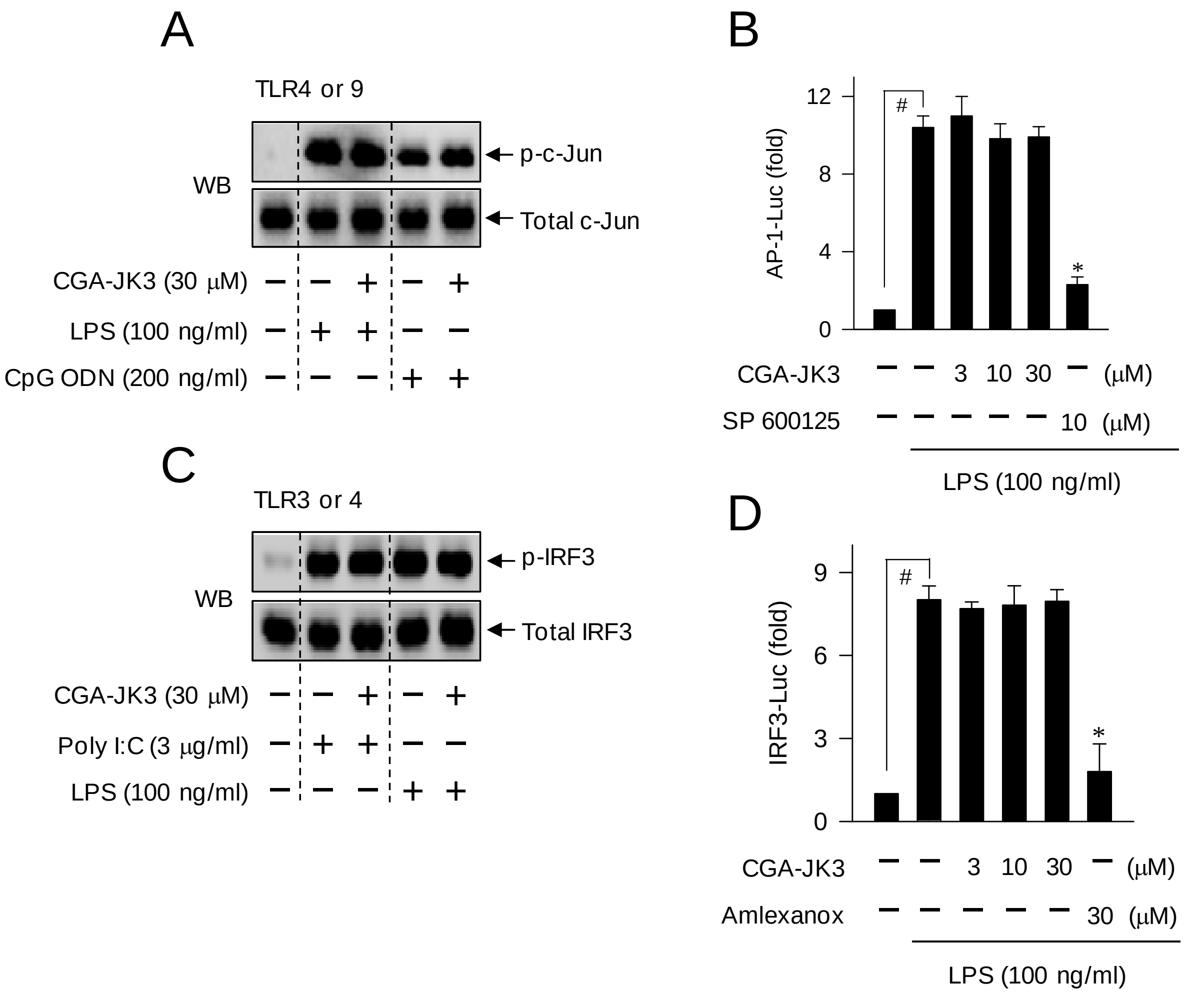

Supplementary Figure 4. Effect of CGA-JK3 on AP-1 or IRF3 activation. RAW 264.7 cells were pretreated with CGA-JK3 for 2 h and stimulated with LPS or CpG ODN (**A**) and poly I:C or LPS (**C**) for 30-40 min in the presence of CGA-JK3. Cell extracts were subjected to Western

blot analysis (WB) with anti-p-c-Jun or anti-c-Jun antibody (**A**) and anti-p-IRF3 or anti-IRF3 antibody (**C**). RAW 264.7 cells were transfected with AP-1-Luc reporter construct (**B**) or IRF3-Luc reporter construct (**D**) in the combination with *Renilla* control vector. The transfected cells were stimulated with LPS for 20 h in the presence of CGA-JK3. Cell extracts were subjected to dual-luciferase assay. Firefly luciferase activity, a reporter of the transcriptional activity of AP-1 or IRF3, is represented as relative fold after normalizing to the *Renilla* activity as a reference of transfection efficiency. Data are mean ± SEM from three independent experiments using the average values of triplicate in each experiment. $^{\#}P < 0.05$ vs. media alone-added group. $^{*}P < 0.05$ vs. LPS alone-stimulated group.

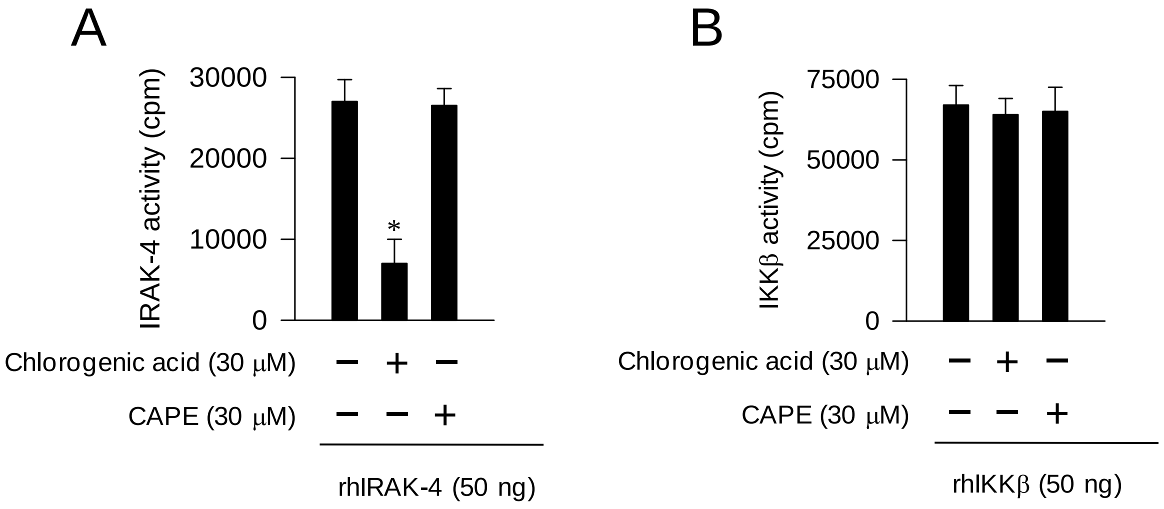

Supplementary Figure 5. Effects of chlorogenic acid and CAPE on the kinase activity of IRAK-4 or IKKβ. Catalytically active rhIRAK-4 (**A**) or rhIKKβ (**B**) was treated with chlorogenic acid or CAPE for 10 min in cell-free reactions. *In vitro* kinase assay was then monitored by the incorporation of [^{32}P] from the probe [γ-^{32}P]ATP onto MBP (**A**) or IKKtide (**B**) as exogenous substrate. Data are mean ± SEM from three independent experiments using the average values of triplicate in each experiment. *P < 0.05 vs. rhIRAK-4 alone-containing group.
